# Preeclampsia and the Risk of Bronchopulmonary Dysplasia in VLBW Infants: A Population Based Study

**DOI:** 10.1371/journal.pone.0075168

**Published:** 2013-09-20

**Authors:** Ting-An Yen, Hwai-I Yang, Wu-Shiun Hsieh, Hung-Chieh Chou, Chien-Yi Chen, Kuo-Inn Tsou, Po-Nien Tsao, for the Taiwan Premature Infant Developmental Collaborative Study Group

**Affiliations:** 1 Department of Pediatrics, National Taiwan University Hospital, National Taiwan University College of Medicine, Taipei, Taiwan; 2 Graduate Institute of Clinical Medical Science, China Medical University, Taichung, Taiwan; 3 Molecular and Genomic Epidemiology Center, China Medical University Hospital, Taichung, Taiwan; 4 Genomics Research Center, Academia Sinica, Taipei, Taiwan; 5 Department of Pediatrics, Cardinal Tien Hospital and College of Medicine, Fu Jen Catholic University, New Taipei City, Taiwan; 6 The Research Center of Developmental Biology and Regenerative Medicine, National Taiwan University, Taipei, Taiwan; University of Liverpool, United Kingdom

## Abstract

**Background:**

Preeclampsia remains a leading cause of maternal mortality and preterm delivery. Both preeclampsia and bronchopulmonary dysplasia (BPD) of prematurity are associated with impaired angiogenesis. However, the relationship between maternal preeclampsia and BPD remains controversial. This study aims to test whether or not preeclampsia is associated with development of BPD in a cohort of premature infants.

**Materials and Methods:**

We conducted a retrospective cohort study assessing the association between preeclampsia and the risk of developing BPD in very-low-birth-weight (VLBW) infants registered in the Premature Baby Foundation of Taiwan from 1997 through 2006. All 21 neonatal departments in Taiwan participated in the data collection. A total of 8,653 VLBW infants were registered in the database. The exclusion criteria included congenital anomalies, chromosome anomalies, infants that died before 36 weeks post-conceptual (PCA), and those whose BPD status were unavailable. BPD was defined as oxygen dependence at 36 weeks postmenstrual age. The association between maternal preeclampsia and BPD was assessed using a multivariate-adjusted logistic regression model.

**Results:**

In the end, a total of 5,753 cases were enrolled in this study. The incidence of preeclampsia was 14.7% (*n*=847) and the overall incidence of BPD was 34.9%. Infants with maternal preeclampsia had a higher gestational age, higher incidence of cesarean section and being small for their gestational age, lower incidence of respiratory distress syndrome, patent ductus arteriosus, and sepsis. BPD occurred significantly less frequently in the maternal preeclampsia group (24.1% vs. 36.7%; adjusted odds ratio: 0.78; 95% confidence interval, 0.62–0.98). Subgroup analysis showed that the association between preeclampsia and BPD was significant only in those VLBW infants with a gestational age between 31–34 weeks.

**Conclusion:**

This data supports the association between fetal exposure to maternal preeclampsia and a reduced risk of BPD in relatively mature VLBW infants.

## Introduction

Preeclampsia results in maternal and fetal morbidity and is a leading cause of preterm delivery [1,2]. The etiology of preeclampsia is not fully understood; however, recent evidence shows that an increase in circulating antiangiogenic factors plays an important role in its pathogenesis [3–6]. Impaired pulmonary vascular growth by altered signaling of angiogenic factors may play a role in the pathogenesis of bronchopulmonary dysplasia (BPD) [7]. Maternal antiangiogenic factors can cross the placenta and may affect angiogenic signaling, thereby altering the risk of BPD [8–13]. Several studies have analyzed the relationship between maternal preeclampsia and the risk of developing BPD in preterm infants [14–25]; however, the results possess a degree of variability and the issue remains controversial.

In this report, we examine the association between preeclampsia and the risk of developing BPD in a large multicenter cohort of very-low-birth-weight (VLBW) infants alive at post-conceptual age (PCA) 36 weeks.

## Materials and Methods

### Study subjects

A total of 8,653 VLBW infants (birth weight less than 1501 g) were born and registered in the database of the Premature Baby Foundation of Taiwan between 1997 and 2006. All 21 neonatal departments in Taiwan participated in the data collection. The data collected included antenatal and perinatal history, infants’ delivery room and neonatal histories including diagnoses, complications during hospitalization, and clinical outcomes at discharge. Patient information received by the database coordinator was cross-checked with the national birth registry. The exclusion criteria included congenital anomalies, chromosome anomalies, infants that died before 36 weeks (PCA), and those whose BPD status were unavailable. Ninety-five infants with maternal chronic hypertension and 38 infants with maternal chronic hypertension with preeclampsia were also excluded. Preeclampsia was defined as a diastolic blood pressure of at least 90 mm Hg accompanied by proteinuria of at least 1+ (30 mg per deciliter) on dipstick testing or nondependent edema during pregnancy [26]. The gestational age (GA) was dated by the last menstrual period or the date of embryo transfer for in vitro fertilization.

### Ethics Statement

The written informed consents were obtained from all their designated relatives. The study was approved by the Institutional Review Boards of each participating hospital, including National Taiwan University Hospital, Chang Gung Memorial Hospital, China Medical University Hospital, National Cheng Kung University Hospital, Tri-Service General Hospital, Chung Shan Medical University Hospital, Shin Kong Wu Ho-Su Memorial Hospital, Kaohsiung Medical University Chung-Ho Memorial Hospital, and Joint Institutional Review Board for the other hospitals.

### Outcome variables

Respiratory distress syndrome (RDS) was defined by clinical diagnosis and requiring surfactant therapy. Necrotizing enterocolitis (NEC) was defined using the criteria of Bell [27], and BPD was defined as oxygen dependence at 36 weeks PCA. We categorized infants as small for gestational age (SGA) if their birth weights were less than the 10th percentile for their gestational ages [28].

### Statistical analysis

The chi-square test and Student’s *t*-test was used for comparing distributions of categorical variables and the continuous variables between groups, respectively. The multivariate logistic regression model was used to analyze the association between maternal preeclampsia and BPD risk adjusted for potential confounders. The confounders include demographic and clinical variables that were different between those with and without preeclampsia by univariate analysis. Adjusted odds ratios with a 95% confidence interval (CI) were derived to assess the magnitude of the association between various factors and BPD risk. Statistically significant levels were determined using the 2-tailed test (*p*<0.05). The association between preeclampsia and BPD was further examined in subgroup analysis with stratification according to GA, SGA, Cesarean section, sex, birth weight, singleton, RDS, patent ductus arteriosus (PDA), and Sepsis.

## Results

A total of 5,753 VLBW infants, including 847 (14.7%) cases born to a mother with preeclampsia, were enrolled. The overall incidence of BPD was 34.9%. Infants born to a mother with preeclampsia were more likely to be of higher gestation, delivered via Cesarean section and being female, small for gestational age and of multiple births. They were also less likely to have RDS, NEC, PDA, and sepsis. The incidence of BPD was significantly lower in infants with maternal preeclampsia compared to those without maternal preeclampsia (24.1% vs. 36.7%; Table 1). The incidence of RDS was significantly lower in the preeclampsia group than in the control group (32% vs. 46%; Table 1; odds ratio (95% CI): 0.55 (0.47–0.64); Table 1). However, after adjusting for GA, BW, SGA, sex, and antenatal steroid usage, preeclampsia was found to have no effect on the incidence of RDS (odds ratio (95% CI): 1.07 (0.87-1.32); Table S1).

**Table 1:** Demographic and clinical variables in infants born to mothers with or without preeclampsia.

**Parameter**	**No preeclampsia**	**Preeclampsia**	***p*-value**
	**N=4906**	**N=847**	
Gestational age^*^	29 (27, 31)	31 (29, 33)	<0.001
Birth weight^*^	1200 (990, 1362)	1198 (990, 1370)	0.8657
Cesarean section^†^ No	2155 (44.2)	68 (8.1)	<0.001
Yes	2726 (55.9)	777 (92.0)	
Sex^‡^ Female	2348 (48.0)	453 (53.7)	0.002
Male	2545 (52.0)	390 (46.3)	
SGA No	3632 (74.0)	226 (26.7)	<0.001
Yes	1274 (26.0)	621 (73.3)	
Singleton^§^ No	1329 (27.2)	124 (14.7)	<0.001
Yes	3561 (72.8)	719 (85.3)	
Antenatal steroid^‖^ < 2 doses	2902 (66.9)	505 (67.0)	0.9727
> 2 doses	1435 (33.1)	249 (33.0)	
RDS^**^ No	2614 (54.0)	564 (68.2)	<0.0001
Yes	2224 (46.0)	263 (31.8)	
NEC^††^ No	4405 (90.0)	780 (92.4)	0.03
Yes	487 (10.0)	64 (7.6)	
PDA^‡‡^ No	3169 (64.9)	631 (75.0)	<0.001
Required treatment	1717 (35.1)	210 (25.0)	
Sepsis^§§^ No	3656 (74.9)	667 (79.3)	0.006
Yes	1228 (25.1)	174 (20.7)	
Days on IPPV	14.6 (26.3)	7.3 (15.3)	<0.001
Days on oxygen, CPAP, or IPPV	41.9 (38.3)	26.3 (29.3)	<0.001
BPD	1802 (36.7)	204 (24.1)	0.001
Duration of hospitalization	73.8 (35.9)	61.8 (27.6)	<0.001

Abbreviations: SGA: small for gestational age; RDS: respiratory distress syndrome; NEC: necrotizing enterocolitis; PDA: patent ductus arteriosus; BPD: bronchopulmonary dysplasia; IPPV: intermittent positive pressure ventilation; CPAP: continuous positive pressure ventilation

^*^ Data presented as median values (25^th^, 75^th^ quartiles)

^†^ A total of 27 subjects were missing for this variable

^‡^ A total of 17 subjects were missing for this variable

^§^ A total of 20 subjects were missing for this variable

^‖^ A total of 662 subjects were missing for this variable

^**^ A total of 88 subjects were missing for this variable

^† †^ A total of 17 subjects were missing for this variable

^‡‡^ A total of 26 subjects were missing for this variable

^§§^ A total of 28 subjects were missing for this variable

In the multivariate logistic regression analysis which included preeclampsia, GA, Cesarean section, sex of baby, birth weight, SGA, singletons, RDS, PDA, and sepsis as risk predictors (Table 2), the preeclampsia was negatively associated with the risk of developing BPD, showing a multivariate-adjusted odds ratio (95% CI) of 0.78 (0.62-0.98). GA, male sex, birth weight, RDS, PDA, and sepsis were also associated with BPD risk. The corresponding odds ratio (95% CI) was 0.83 (0.78–0.88), 1.47 (1.28–1.68), 0.78 (0.74–0.82), 3.05 (2.65–3.52), 1.21 (1.05–1.39), and 1.43 (1.23–1.67), respectively (Table 2).

**Table 2:** Multivariate-adjusted odds ratio of developing BPD for various factors.

**Parameter**		**Odds ratio (95% CI)**	***p*-value**
Preeclampsia (yes vs. no)		0.78 (0.62,0.98)	0.03
Gestational age		0.83 (0.78,0.88)	<.001
Cesarean section (yes vs. no)		0.93 (0.80–1.08)	0.31
Sex of baby (male vs. female)		1.47 (1.28,1.68)	<.001
Birth weight (per 100 grams)		0.78 (0.74,0.82)	<.001
SGA (yes vs. no)		1.02 (0.79,1.30)	0.90
Single (yes vs. no)		1.13 (0.96,1.33)	0.15
RDS (yes vs. no)		3.05 (2.65,3.52)	<.001
PDA (required treatment vs. no)		1.21 (1.05,1.39)	0.01
Sepsis (yes vs. no)		1.43 (1.23–1.67)	<.001

Abbreviations: SGA: small for gestational age; RDS: respiratory distress syndrome; PDA: patent ductus arteriosus

**Figure 1:**
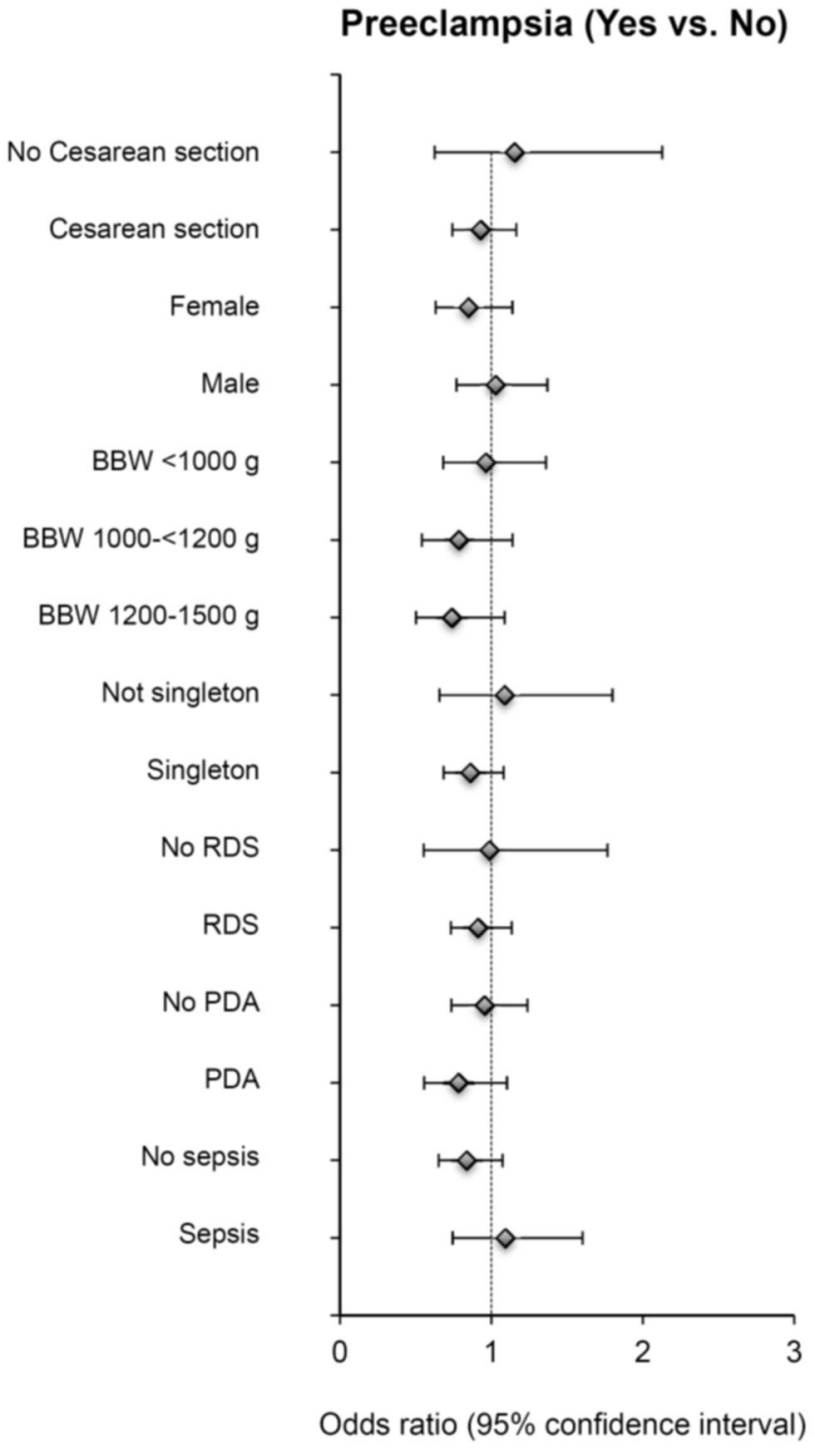
Subgroup analyses for the association between preeclampsia and BPD. Odds ratios were adjusted for sex, GA (asa continuous variable), RDS, and SGA, except for the stratifying variables.

Since GA and SGA are two known factors that are associated with the risk of BPD in preterm infants, we further performed the subgroup multivariate-adjusted analysis with stratification according to GA groups, SGA status, cearean section, sex, groups of body weight, singleton, RDS, and PDA. We observed a statistically significant association between preeclampsia and BPD in those VLBW infants with GA greater than 31 weeks (adjusted-OR, 0.51; 95% CI, 0.30–0.88), but not in other GA groups (Table 3). When we separated these VLBW infants into SGA and non-SGA groups, maternal preeclampsia decreased the risk of developing BPD only in SGA group (adjusted-OR, 0.64; 95% CI, 0.46–0.90) but not in non-SGA group (Table 4). We did not find a statistically significant association between maternal preeclampsia and BPD risk in all other subgroups (Figure 1).

**Table 3:** Multivariate-adjusted odds ratio of BPD for preeclampsia with stratification according to GA.

**Preeclampsia**	**GA: ≤27**		**GA: 27–29**		**GA: 29–31**		**GA: 31–34**	
	**n**	**BPD n (%)**	**n**	**BPD n (%)**	**n**	**BPD n (%)**	**N**	**BPD n (%)**
No	1407	955 (67.9)	1455	524 (36.0)	1210	249 (20.6)	658	71 (10.8)
Yes	75	52 (69.3)	162	78 (48.2)	248	55 (22.2)	299	18 (6.0)
OR (95% CI)^*^	0.96 (0.56–1.66)		1.13 (0.78–1.63)		0.98 (0.68–1.40)		0.51 (0.30–0.88)	
*p*-value	0.886		0.510		0.898		0.016	

Abbreviations: GA: gestational age; BPD: bronchopulmonary dysplasia; OR: odds ratio; CI: confidence interval

* OR is adjusted for sex, GA, RDS and SGA.

**Table 4:** Multivariate-adjusted logistic regression analysis of BPD development with stratification according to SGA.

**Parameter**	**SGA**		**Non-SGA**	
	**Odds ratio (95% CI)**	***p*-value**	**Odds ratio (95% CI)**	***p*-value**
Preeclampsia (yes vs. no)	0.64 (0.46,0.90)	0.01	0.85 (0.59,1.23)	0.387
Gestational age	0.70 (0.63,0.77)	<.0001	0.58 (0.53,0.64)	<.001
Birth weight (per 100 grams)	0.70 (0.64,0.77)	<.0001	0.76 (0.70,0.82)	<.001
Cesarean section (yes vs. no)	0.65 (0.44,0.95)	0.0253	1.01 (0.84,1.21)	0.944
Sex (male vs. female)	1.61 (1.19,2.19)	0.0022	1.32 (1.10,1.58)	0.002
Single (yes vs. no)	1.00 (0.71,1.40)	0.9944	1.16 (0.94,1.44)	0.1645
RDS (yes vs. no)	4.38 (3.16,6.06)	<.0001	3.07 (2.57,3.66)	<.001
Patent ductus arteriosus (yes vs. no)	2.15 (1.52,3.05)	<.0001	1.17 (0.97,1.41)	0.092
Sepsis (yes vs. no)	1.36 (0.95,1.94)	0.0924	1.36 (1.10,1.67)	0.004

Abbreviations: SGA: small for gestational age; CI: confidence interval; RDS: respiratory distress syndrome

When we focused on those extremely preterm infants with a gestational age <28 weeks or birth weight <1000 gm, we found that 11.8% (227/1924) of the infants fell into the preeclampsia group; the incidence of BPD was similar in preeclampsia and control group (56% (127/227) vs. 64% (1083/1697); OR (95% CI): 0.96 (0.67–1.39)). This data indicates that there is no association between preeclampsia and BPD in extremely preterm or extremely-low-birth-weight infants who are at higher risk of developing BPD.

## Discussion

In this population-based large cohort study of VLBW infants, we found in overall Taiwanese infants, maternal preeclampsia to be an independent factor associated with a decreased risk of BPD (Table 2). However, this negative association was only valid in subgroup of VLBW infants with GA greater than 31 weeks and in SGA subgroup. We did not observe a statistically significant association in extremely preterm infants with GA < 28 weeks or birth weight < 1000 gm.

RDS is one of the most common diseases in preterm infants and is also a leading cause of subsequent BPD [29]. The relationship between preeclampsia and RDS is controversial [30–36]. The discrepancy among these studies may in part be result from relatively small sample sizes. Recently, Langenveld et al. demonstrated that the incidence of RDS was reduced in late preterm infants born to mothers with preeclampsia [37]. In our study, we also found that VLBW infants with maternal preeclampsia were less likely to develop RDS than those without maternal preeclampsia (Table 1). However, this protective effect disappeared after adjustment for confounding variables (Table 1). RDS usually appeared in VLBW infants but not in late preterm neonates. The diagnosis of RDS is mainly based on chest radiography as Langenveld et al. did, thus the incidence in different reports may vary. To minimize the bias of diagnosis of RDS, we defined the RDS as VLBW infants with RDS who required surfactant therapy. Our data suggest that preeclampsia may not be associated with the risk of RDS in VLBW infants when GA, BW, SGA, sex, and antenatal steroid usage were taken into consideration.

Previously, Hansen et al. and Korhonen et al. reported that maternal preeclampsia was an independent risk factor for BPD in multivariate analysis (odds ratio (95% CI): 18.7% (2.44–144.76) and 6.75% (1.22–37.3), respectively) [17,19]. Kurkinen-Räty et al. and Withagen et al. had similar findings as well [20,24]. However, Schlapbach et al., Cheng et al., Cetinkaya et al. and O’Shea et al. did not find this association [15,16,21,23]. In addition, Redline et al. studied the placenta and perinatal risk factors for BPD in VLBW infants [22]. Although no relationship between clinical preeclampsia and BPD was observed, placental acute atherosis (a placental indicator of preeclampsia) was found to be inversely related to BPD (7% vs. 12%; odds ratio, 0.2; 95% CI, 0.1–0.8). Recently, Hansen et al. demonstrated that BPD increased in infants exposed to preeclampsia and hypothesized that this may due to the maternal antiangiogenic state. However, their sample size is small. In this large multicenter study, we provided the evidence that maternal preeclampsia was associated with a decreased risk of BPD in overall VLBW infants. Nonetheless, with the large sample size, we were able to examine this association in depth and demonstrated that this protective effect was only seen in particular subgroups.

In agreement with the report from O’Shea et al. [21], we found that preeclampsia does not affect the risk of BPD in extremely preterm or ELBW infants. In addition, we found that GA is a consistent independent risk factor of BPD in all our analyses, as was expected. This indicates that prematurity itself has a very important independent influence on developing BPD and this effect is not affected by preeclampsia (Table 3). Interestingly, when we performed subgroup analysis, the protective effect of maternal preeclampsia was found to be significant in a relatively mature group (GA: 31–34 weeks), especially for those without RDS. Our data showed that RDS and prematurity as indicated by the GA were both significantly and independently associated with BPD (Table 2). The extreme prematurity and RDS may have a very profound effect on the development of BPD to some extent that they mask the potential protective effect of preeclampsia. Conversely, the effect of preeclampsia was much more evident in a more mature preterm infant (31–34 weeks gestation), as shown in our study.

Intrauterine growth restriction or SGA has been reported to be associated with both preeclampsia and BPD [12–14,16,38–44]. The protective effect of preeclampsia was only found in SGA, but not non-SGA, preterm infants in our report. The reason for this difference is unclear. One possible explanation is that the severity of preeclampsia is usually strongly negatively associated with fetus body weight. This may explain why the protective effect of preeclampsia was absent in the non-SGA group.

The mechanism of preeclampsia that protects VLBW infants from BPD is not clear. Increasing circulating soluble Flt-1, a soluble form of vascular endothelial growth factor (VEGF) receptor-1 which can bind both VEGF and placenta growth factor (PGF), resulting impairment of angiogenic state was thought to be responsible for the pathogenesis of preeclampsia [3–6,45–47]. In developing lungs, disruption of VEGF signaling impaired angiogenesis decreased alveloarization [8,10,48]. Although the cord blood levels of sFlt-1 was also elevated in preeclampsia group [9,12], but the levels were significantly lower than maternal levels [9]. This resulted in only mild or no significant difference in VEGF levels between preeclampsia and control groups can be found [9,12]. Similarly, cord PGF levels were also lower in preterm infants born to mothers with preeclampsia [12]. In contrast to VEGF, elevated PGF expression contributed pulmonary emphysema in mouse model and increased risk of developing BPD in preterm infants [11,13]. Therefore, preeclampsia-decreased PGF levels in cord blood may possibly contribute to the protective effect vis-à-vis developing BPD in preterm infants [49].

The strength of our study was that it was a large multicenter cohort study, allowed us to assess the association between maternal preeclampsia and BPD in several subgroup analyses. Our study also had some limitations. First, the reliability of our data depended on the preciseness of pediatricians and case managers. Second, some data of interest were unavailable in some cases. However, the large size of this database and nondifferential misclassification minimized these influences. Third, the strategies of respiratory care differed among different hospitals. This indeed influences the overall incidence of BPD, but may not lead to bias because the strategy should be the same regardless of whether preterm infants were born to mothers with or without preeclampsia in the same hospital. Fourth, our cohort was defined by birth weight. However, because this could have caused overrepresentation of growth-retarded infants, we performed several subgroup analyses to avoid this problem. Finally, the definition of BPD used here is a traditional clinical definition but not a physiological definition, a discrepancy which reduced the overall rate of BPD and reduced the variation among centers [50]. However, this cohort started as early as 1997 when there were no criteria for physiological definition of BPD.

In conclusion, our data supports the association between fetal exposure to maternal preeclampsia and a reduced risk of BPD in the particular subgroup of VLBW infants with gestational age greater than 31 weeks.

## Supporting Information

Table S1 **The regression analyses on factors associated with RDS.**
(DOC)
