# Supplementary material for: Preeclampsia and the Risk of Bronchopulmonary Dysplasia in VLBW Infants: A Population Based Study
Source: PLoS One. 2013 Sep 20;8(9):e75168. doi: 10.1371/journal.pone.0075168 (PMC3779258; doi:10.1371/journal.pone.0075168)
Supplement: Table S1 — The regression analyses on factors associated with RDS. (DOC) [file pone.0075168.s001.doc]

**Table S1.** The regression analyses on factors associated with RDS

| **Variable** | **Univariate model** | | **Multivariate model 1** | | **Multivariate model 2** | |
| --- | --- | --- | --- | --- | --- | --- |
|  | **Odds ratio (95% CI)** | **P value** | **Odds ratio (95% CI)** | **P value** | **Odds ratio (95% CI)** | **P value** |
| GA | 0.64 (0.62-0.66) | <.0001 | 0.69 (0.65-0.73) | <.0001 | 0.66 (0.64-0.69) | <.0001 |
| Birth weight (per 100 grams) | 0.72 (0.71-0.74) | <.0001 | 1.00 (1.00-1.00) | 0.0003 | 1.00 (1.00-1.00) | 0.002 |
| Sex of baby (male vs. female) | 1.33 (1.20-1.48) | <.0001 | 1.33 (1.16-1.52) | <.0001 | 1.29 (1.14-1.47) | 0.0001 |
| SGA (yes vs. no) | 0.32 (0.28-0.36) | <.0001 | 0.78 (0.62-0.99) | 0.0418 | (not included) |  |
| Preeclampsia (yes vs. no) | 0.55 (0.47-0.64) | <.0001 | 1.07 (0.87-1.32) | 0.5139 | 1.04 (0.85-1.27) | 0.7361 |
| Prenatal steroid (yes vs. no) | 0.74 (0.66-0.84) | <.0001 | 0.59 (0.51-0.68) | <.0001 | 0.59 (0.51-0.68) | <.0001 |

SGA= small for gestational age
